# Performance of spectral flow cytometry and mass cytometry for the study of innate myeloid cell populations

**DOI:** 10.3389/fimmu.2023.1191992

**Published:** 2023-05-19

**Authors:** Kyra van der Pan, Indu Khatri, Anniek L. de Jager, Alesha Louis, Sara Kassem, Brigitta A.E. Naber, Inge F. de Laat, Marjolijn Hameetman, Suzanne E.T. Comans, Alberto Orfao, Jacques J.M. van Dongen, Paula Díez, Cristina Teodosio

**Affiliations:** ^1^ Department of Immunology, Leiden University Medical Center (LUMC), Leiden, Netherlands; ^2^ Flow Cytometry Core Facility, Leiden University Medical Center (LUMC), Leiden, Netherlands; ^3^ Translational and Clinical Research Program, Cancer Research Center (IBMCC; University of Salamanca - CSIC), Cytometry Service, NUCLEUS, Department of Medicine, University of Salamanca and Institute of Biomedical Research of Salamanca (IBSAL), Salamanca, Spain; ^4^ Sarcomas and Experimental Therapeutics Laboratory, Health Research Institute of Asturias (ISPA) and Asturias Central University Hospital (HUCA), Department of Physical and Analytical Chemistry, Faculty of Chemistry, University of Oviedo, Oviedo, Asturias, Spain

**Keywords:** spectral flow cytometry, myeloid cells, immunophenotyping, cyTOF, mass cytometry

## Abstract

**Introduction:**

Monitoring of innate myeloid cells (IMC) is broadly applied in basic and translational research, as well as in diagnostic patient care. Due to their immunophenotypic heterogeneity and biological plasticity, analysis of IMC populations typically requires large panels of markers. Currently, two cytometry-based techniques allow for the simultaneous detection of ≥40 markers: spectral flow cytometry (SFC) and mass cytometry (MC). However, little is known about the comparability of SFC and MC in studying IMC populations.

**Methods:**

We evaluated the performance of two SFC and MC panels, which contained 21 common markers, for the identification and subsetting of blood IMC populations. Based on unsupervised clustering analysis, we systematically identified 24 leukocyte populations, including 21 IMC subsets, regardless of the cytometry technique.

**Results:**

Overall, comparable results were observed between the two technologies regarding the relative distribution of these cell populations and the staining resolution of individual markers (Pearson’s ρ=0.99 and 0.55, respectively). However, minor differences were observed between the two techniques regarding intra-measurement variability (median coefficient of variation of 42.5% *vs.* 68.0% in SFC and MC, respectively; p<0.0001) and reproducibility, which were most likely due to the significantly longer acquisition times (median 16 min *vs.* 159 min) and lower recovery rates (median 53.1% *vs.* 26.8%) associated with SFC *vs.* MC.

**Discussion:**

Altogether, our results show a good correlation between SFC and MC for the identification, enumeration and characterization of IMC in blood, based on large panels (>20) of antibody reagents.

## Introduction

Immune profiling of granulocytes, monocytes and dendritic cells (DC), also known as innate myeloid cells (IMC), provides detailed information about normal homeostatic conditions and multiple disease conditions (1–3). It is well known that IMC display a high phenotypical and functional plasticity. Such heterogeneity, together with the expression of a limited set of lineage-specific proteins and the existence of an increasing number of (recently described) IMC subsets (4–8), demands large sets of markers for accurate immunophenotypic identification and characterization of IMC.

At present, simultaneous evaluation of large panels (≥40) of IMC-associated markers in blood leukocytes can be achieved in practice *via* two different approaches: mass cytometry (MC) and spectral flow cytometry (SFC) (9, 10). In MC, also known as cytometry by time-of-flight or CyTOF, antibodies tagged with distinct heavy metal isotypes instead of fluorochromes are used for the detection of different markers on individual cells. Such an approach overcomes the constraints of spectral overlap in conventional flow cytometry (11), since the antibody-labelled cells are detected by measuring the mass of ions and are separated for each heavy metal based on its specific mass-to-charge ratios of the resulting ions (12, 13). Accordingly, MC allows for simultaneous assessment of >50 markers, but cannot provide information about the cell size, internal complexity and autofluorescence profile. Of note, high levels of autofluorescence are usually considered a drawback in flow cytometry, although it can provide useful additional information concerning specific IMC populations (14). Recently, SFC was shown to be an alternative approach to MC, based on the possibility to apply large panels of (≥40) markers, particularly in high-end ≥5-laser instruments (10, 15). SCF allows for the simultaneous assessment of the complete emission spectrum of the applied fluorochromes, after excitation by multiple lasers (10, 16). Once the primary fluorescence signals are deconvoluted by mathematical algorithms, the individual spectral signatures of fluorochromes can be identified within a mixture of many different fluorochromes (12). Overall, MC currently allows for simultaneous measurement of a higher number of markers (>50) conjugated with different labels, compared to SFC, due to the availability of more unique (i.e. compatible) heavy metal labels (13, 17). However, SFC has higher sensitivity for the detection of low-abundant proteins (≈40 *vs.* 400-500 molecules per cell in SFC and MC, respectively) (12), and a greater speed of analysis of thousands (≈20,000) events per second *vs* ≈300 events per second for SFC and MC, respectively (17).

So far, several studies compared the performance of SFC *vs.* MC for immune cell monitoring, with an overall good concordance (correlation coefficient >0.98) (12, 18, 19). However, these studies have evaluated murine cells (18) that show less inter-donor variation compared to human samples (20), they did not address infrequent cell populations (12, 19), eliminated day-to-day instrument-related variation by measuring all samples on the same day (12) and/or they focused on lymphoid cell populations, with limited attention for IMC populations (12, 18, 19).

In most of the above-cited studies, lymphoid cells have been extensively evaluated, while identification of IMC populations is more complex and cannot straightforwardly rely on specific lineage markers, but is merely based on differential expression levels of (often) co-expressed proteins [e.g., HLA-DR (21), CD33 (22), CD5 (23) and CD14/CD16 for monocytic subsets (24–26)]. Furthermore, several IMC populations are present at very low frequencies (<0.01% of all leukocytes) in the blood of healthy human subjects (27) (e.g., Axl+ DC and CD141+ myeloid DC). In this context, the lower sensitivity of MC, and the potential spread associated with fluorescence-based SFC, might hamper the design of high-sensitive >20 marker panels for robust identification and characterization of human IMC. This might explain the poor correlation (correlation coefficient = 0.27) between both technologies, previously reported in the few studies addressing myeloid cells (12).

Moreover, monitoring of IMC in diagnostic laboratories frequently requires fast parallel analysis of multiple patient samples with short turnaround times. Therefore, it is not an option to have storage of patient samples for subsequent batch-based processing and analysis. In addition, storage of IMC by freezing is known to have an impact on marker epitopes and cell viability (27). Consequently, there is a need for highly-reproducible assays across different days, with minimal over-time variations, which would hamper the comparability of the acquired data. Several mathematical methods have been developed to correct for technical variation of sample analysis at different time points, using parallel processing of a reference sample (i.e., a common sample processed and analyzed in parallel to the test sample) (28–31). Such strategies cannot be easily implemented in the daily routine workflow of clinical laboratories.

Here, we compared the use of SFC and MC for studying circulating IMC populations based on in-house developed antibody panels, aimed at a potential implementation in clinical laboratories. We ultimately compared the performance of SFC *vs.* MC concerning identification, characterization and enumeration of human blood IMC populations, together with the inter- and intra-assay variability.

## Materials and methods

### Sample collection and processing

Fresh citrate-anticoagulated peripheral blood (PB) samples were collected from five healthy adult volunteers (median age 28, range 25-31; female/male ratio of 2/3) from the Sanquin Bloedvoorziening (Amsterdam, The Netherlands) under the research project code NVT0532.01. All donors provided their informed consent to participate in the study according to the Declaration of Helsinki and the guidelines of the local ethics committee. Within 2h after collection, PB mononuclear cells (PBMC) were isolated by Ficoll-Paque Plus gradient centrifugation (GE Healthcare Bio-Sciences AB, Uppsala, Sweden), according to the manufacturer’s instructions, as described elsewhere (27). After isolation, PBMC were washed (5min at 520 g) and resuspended in phosphate-buffered saline (PBS; Lonza, Basel, Switzerland). Subsequently, PBMC were counted using a Sysmex XP-300 automated hematological analyzer (Sysmex Europe GmbH, Norderstedt, Germany), and 5x10^6^ PBMC were used for staining with the SFC and MC antibody panels.

### Antibody selection

Two antibody panels specifically designed for the study of IMC populations were used to stain PBMC, based on previous reports (EuroFlow patent “Means and methods for multiparameter cytometry-based leukocyte subsetting”; PCT/NL2020/050688, priority date 5 November 2019) (27, 32–36). Briefly, the selection of the markers employed in both MC and SFC combinations was performed based on unbiased identification of the most optimal set of markers to identify each population, in order to provide the best population discrimination and avoid any type of redundancy (27, 37–41). These included a 33-marker MC combination, aimed at the study of IMC across multiple tissues, and a 24-marker panel, designed for the study of PB and bone marrow samples employing CE-IVD-certified SFC instruments (e.g., 3 laser Cytek Northern Lights™) (**Supplementary Table 1**). A total of 21 markers were shared between both SFC and MC panels (**Supplementary Table 1**), for which the same clones were used in both platforms whenever possible (15/21 markers). For the other 6/21 markers, different clones were used due to lack of availability or poor performance of the antibody clone in one of the platforms. Prior to use, all antibody reagents were titrated for optimal signal-to-noise ratios, according to EuroFlow guidelines (42). The level of expression of each marker and its expression profile were assessed for each of the different target IMC populations, prior to panel design.

### Mass cytometry panel design, sample processing and data acquisition

Since mass cytometers are most sensitive for metals in the 159-175 mass range (13), dimly expressed markers were evaluated using heavy metals within this range. In addition, isotopic impurities (43, 44) causing spillover in adjacent channels, as well as spillover signals caused by oxidation (+16 Da) (43, 44) were minimized by placing markers not co-expressed by the same cells in these channels.

Due to the lack of commercial availability of the most optimal antibody-heavy metal combinations for the panel, in-house conjugation was performed for the majority of the markers (20/21) employing 100 µg of carrier-, glycerol- and BSA-free purified IgG antibodies and the MaxPar^®^ X8 Antibody Labeling Kit, according to the manufacturer’s instructions (Standard Biotools, San Francisco, CA), as described in detail in **Supplementary Material** and **Supplementary Methods**.

PBMC were stained immediately after isolation. Briefly, isolated PBMC were washed with 2 mL of cold Cell Staining Buffer (CSB; Standard Biotools) and centrifuged at 500 g for 5 min, resuspended in 1 mL of CSB supplemented with 1 µM Intercalator-Rh (Standard Biotools) and incubated for 15 min at room temperature (RT). Afterwards, the cell suspension was washed with 1 mL of CSB, centrifuged for 5 min at 500 g, and resuspended in 45 µL CSB, after discharging the supernatant. Subsequently, PBMC were incubated with 5 µL of Fc-receptor blocking solution (Human TruStain FcX™; Biolegend, San Diego, CA) for 10 min, and incubated with 50 µL of heavy-metal conjugated antibodies (**Supplementary Table 1**) for another 45 min in agitation on a plate shaker at RT. Then, stained PBMC were washed three times with 2 mL of CSB (500 g 5 min) and incubated with 1 mL of the Maxpar^®^ Fix and Perm Buffer (Standard Biotools) supplemented with 0.125 µM Intercalator-Ir (Standard Biotools) for 1 h at RT. Afterwards, stained PBMC were washed (3 times at 800 g for 5 min) with CSB, resuspended in 500 µL of MilliQ water and counted in a Neubauer chamber. Shortly before acquisition, cells were washed twice and resuspended in MilliQ water in a concentration of 0.7x10^6^ cells/mL. EQ™ Four Element Calibration Beads (Standard Biotools) were added at a 1:10 ratio, for measurement normalization, prior to the measurement of the samples on a Helios mass cytometer (Standard Biotools). Instrument set-up was performed strictly following the recommendations of the manufacturer, as described elsewhere (12). Data was acquired employing the default settings and signal fluctuations were normalized with the reference EQ passport EQ4_P13H2302. To control for technical variation in sample preparation, a common reference sample (containing positive reference populations for each marker included in the panel) was processed and measured in parallel to each test sample.

Correction for potential spillover due to detection sensitivity, isotopic impurities, and oxide formation was performed using the Shiny-based web version of CATALYST (Cytometry dATa anALYSis Tools), CatalystLite (45). Single stained MACS^®^ Comp Beads (Miltenyi Biotec, Bergisch Gladbach, Germany) or OneComp eBeads™ Compensation Beads (Thermo Fisher, Waltham, MA) (depending on whether the antibody was recombinant or not), stained according to the manufacturer’s instructions were used as reference beads for spillover calculation as described in detail in the **Supplementary Materials** and **Supplementary Methods section**.

### Spectral flow cytometry panel design, sample processing and data acquisition

Panel design for SFC was performed following previously described recommendations (10, 12). Briefly, fluorochromes were selected based on their unique spectral signatures when run in a 3-laser (405nm, 488nm, 640nm) Aurora (Cytek, Fremont, CA) instrument. Fluorochrome-antibody combinations were selected based on brightness and spread (10, 46). Thus, whenever possible, strongly expressed markers with a high degree of co-expression with other markers in the same population(s) were assigned to dimmer fluorochromes, to ensure optimal resolution and minimal spread. Conversely, markers expressed at low levels in the target cell populations were paired with bright fluorochromes. Prior to the evaluation of the tested samples, the performance of the designed panel was validated to ensure accurate identification of the cell populations of interest.

Sample staining was performed following the EuroFlow staining standard operating procedures (SOP) available at www.EuroFlow.org. Briefly, PBMC were resuspended in washing buffer (PBS containing 0.5% bovine serum albumin (BSA), 0.1% sodium azide and 2 mM ethylenediaminetetraacetic acid (EDTA); pH=7.4) and incubated on a roller with the antibodies in the presence of Brilliant Staining Buffer Plus (BD Biosciences, San Jose, CA) (**Supplementary Table 1**) for 30 min at RT (protected from light). Afterwards, the stained samples were washed with PBS (5 min at 500 g) and incubated with a viability marker (Zombie NIR, Biolegend) in a 1:1000 dilution for 30 min at RT, protected from light. To prevent potential contamination with erythrocytes and to fix the cells, samples were subsequently incubated with 1x BD FACS Lysing Solution (BD Biosciences) for 10 min at RT (protected from light), centrifuged for 5 min at 500 g, washed with washing buffer and resuspended in 500 µL of PBS before analysis in the SFC. Data acquisition was performed at a medium flow rate (30 µL/min) on a 3-laser (405nm, 488nm, 640nm) Aurora (Cytek). Prior to data acquisition, daily instrument set-up and quality control were performed following the manufacturer’s instructions. For each fluorochrome in the antibody combination, single-stained reference controls, as well as an unstained control sample (per donor), were processed strictly following the same procedure as used for the multicolor-stained samples, and measured prior to the acquisition of the test sample(s), to ensure correct spectral unmixing. The resulting unmixing matrix created by the SpectroFlo v2.2.0 software (Cytek), was applied for each test sample after it had been measured using the live unmixing function.

### Data quality control and analysis

Clean-up of the MC files was performed as previously described in detail using Infinicyt™ software (version 2.0.2.d.000; Cytognos S.L., Salamanca, Spain) (12). Briefly, gating on 191Ir and 140Ce was first done to identify the cells separately from the EQ™ Four Element Calibration Beads, followed by elimination of doublets, based on Intercalator-Ir (191Ir/193Ir) signal and event length patterns. Signal stability was evaluated by plotting time of acquisition *vs.* CD45; instability of acquisition observed for the data collected at the beginning of the measurement (detected in 2/5 samples) was excluded from further analysis. Live leukocytes were further selected based on a CD45^+^, Intercalator-Ir^+^ and Intercalator-Rh^-^ expression profile and gated data exported for further analysis.

Quality control of SFC data was also performed using Infinicyt™ as described elsewhere (10, 12). Briefly, debris and dead cells were removed based on the expression of the CD45 signal and the viability marker (Zombie NIR). The stability of the signal during acquisition was evaluated by plotting Time *vs.* CD45, and singlets were identified on a conventional forward scatter -(FSC) Area *vs.* FSC-Height dot plot and gated data exported for further analysis. Additionally, NxN plots were used to evaluate the unmixing accuracy in the multicolor tubes (10), and when necessary, appropriate corrections were applied, the largest correction corresponding to -9% for the PE Cy7 signal in the Qdot800 channel.

Unsupervised analysis of SFC and MC data files was performed using FlowJo™ software (v10, BD Biosciences), based on the Uniform Manifold Approximation and Projection for Dimension Reduction (UMAP) (47) and FlowSOM (48) plugins for both data clustering and visualization. To avoid potential bias in the comparison between the two platforms, due to the inclusion of additional parameters for population clustering, data on scatter parameters (FSC and sideward light scatter – SSC-), available in the SFC files, was excluded from further analyses.

In a first step, identification of the major cell populations per lineage within PBMC was performed. T cells were identified as CD5^+^ CD16^-^ CD33^-^ HLA-DR^-/lo^; B cells as CD5^-/lo^ CD16^-^ CD33^-^ HLA-DR^hi^ cells; NK cells as CD5^-^ CD16^+^ CD33^-/lo^ HLA-DR^-/lo^ cellular events; basophils as FcϵRI^+^ HLA-DR^-^ cells; and the remaining myeloid cell compartment as HLA-DR^hi^ CD33^+^ and/or CD303^+^ and/or CD34^+^ cells (**Supplementary Figure 1**). Subsequently, the myeloid cell compartment was investigated in more detail, to identify all minor myeloid (sub)populations. FlowSOM was employed for further clustering of cells with over clustering ≥ 7 times more clusters than expected cell populations to ensure the identification of rare subsets. Whenever clusters were identified which consisted of multiple subsets, additional clustering was performed. Identification of the different IMC (sub)populations was done according to previously described immunophenotypic patterns (**Supplementary Figures 2**, **3** exemplify the overall immunophenotypic profiles employed as well as the manual gating strategy traditionally employed to identify the populations) (27).

### Statistical methods

The resolution of each individual marker was calculated based on their average overlap frequency (AOF) using its corresponding positive and negative reference population (PRP and NRP), respectively (R 4.1.0; R Core Team, 2021), as described elsewhere (49) (**Supplementary Table 2**). Median, range, mean, standard deviation (SD) and % coefficient of variation (CV) values, as well as the 10^th^, 25^th^, 75^th^ and 90^th^ percentiles, were calculated for continuous variables. The degree of association between two continuous variables was determined employing the Pearson’s correlation. Statistical significance (p-value <0.05) of differences between groups was determined (for continuous variables) by the Mann-Whitney test or the non-parametric Kruskal-Wallis and *post hoc* Dunn’s multiple comparisons tests (for independent samples) and the Wilcoxon matched-pairs signed rank test (for paired samples), whereas the Fisher’s exact test was employed for categorical data. For all statistical analyses, GraphPad Prism (version 9.3.1) (GraphPad, San Diego, CA) was used.

## Results

### Identification and quantification of myeloid populations by SFC *vs.* MC

Globally, MC showed a significantly lower median (range) rate of recovery (p=0.008) of PBMC compared to SFC: 26.8% (23.2% - 31.7%) *vs.* 53.1% (40.4% - 64.4%), respectively. To avoid a bias due to the evaluation of distinct numbers of PBMC, analysis of SFC samples was also performed on randomly selected cells from down sampled files (to the same number of live cellular events as measured in the corresponding MC samples). From here on, these data files are referred to as down sampled SFC data files/samples (dSFC).

**Figure 1:**
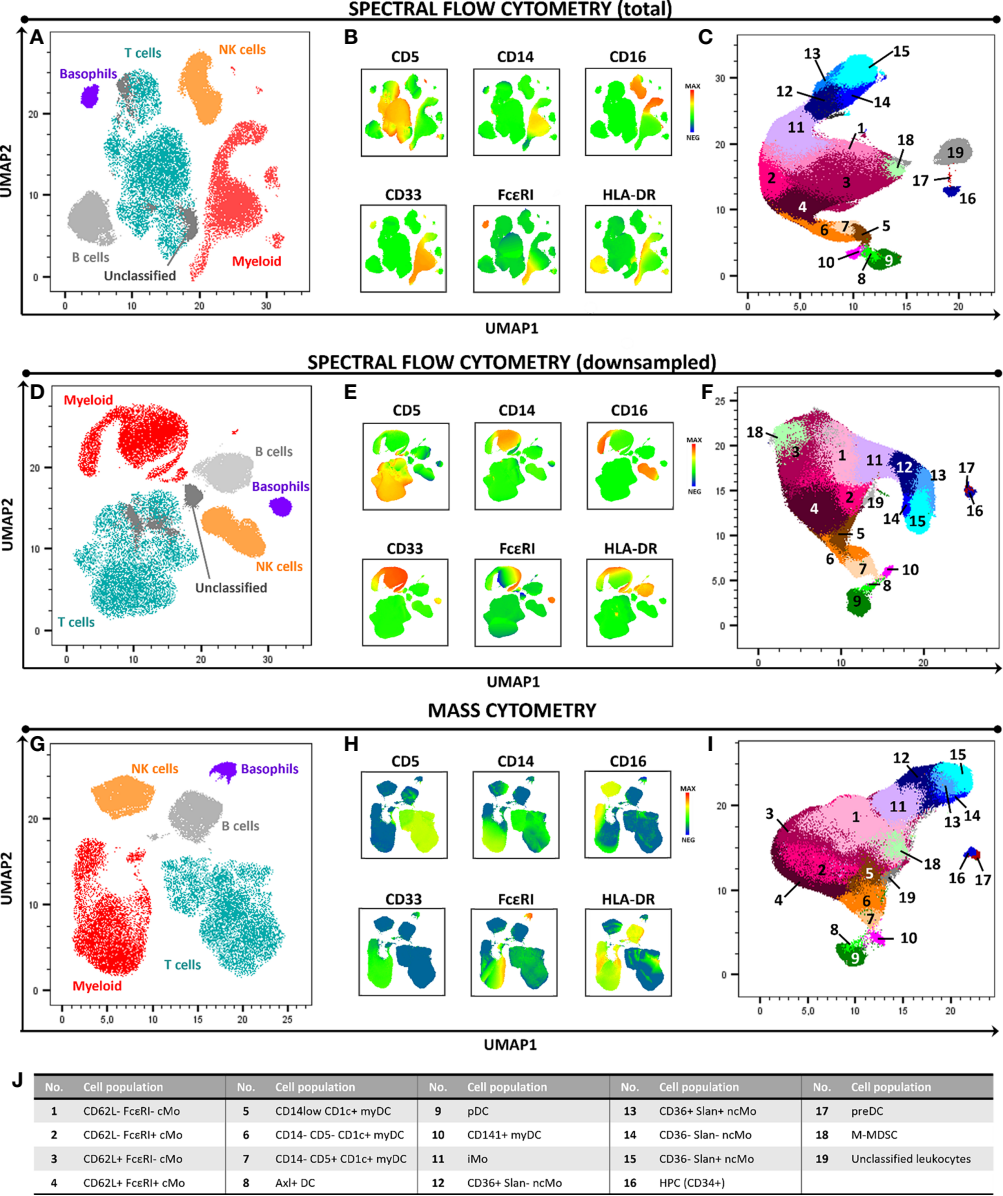
High-dimensional 2D graphical representation of paired peripheral blood mononuclear cell (PBMC) samples analyzed by spectral flow cytometry (SFC) and mass cytometry (MC), based on a combination of 21 markers. Major cell populations and the immunophenotypic expression patterns used for their identification are shown in panels **(A, B, D, E, G, H)**. In-depth dissection of the myeloid cell compartment (excluding basophils) is depicted in panels **(C, F, I, J)**. In panels **(A–C)** and **(D–F)** samples were evaluated by SFC (full sample and downsampled to match the number of cells collected by MC, respectively). Panels **(G–I)** depict the profile of a paired sample analyzed by MC. *Axl DC, Axl^+^ dendritic cell; cMo, classical monocyte; HPC, hematopoietic precursor cell; iMo, intermediate monocyte; myDC, myeloid dendritic cell, M-MDSC, monocytic myeloid-derived suppressor cell; ncMo, non-classical monocyte; pDC, plasmacytoid dendritic cell; preDC, CD100^+^ dendritic cell precursor*.

**Figure 2:**
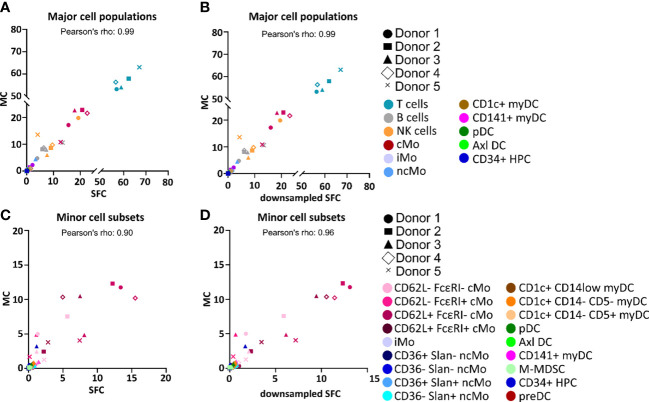
Correlation between the percentage values of individual cell populations among all peripheral blood mononuclear cells (PBMC) as analyzed by spectral flow cytometry (SFC) *vs.* mass cytometry (MC). Correlation between the relative frequency of different populations of PBMC detected in paired samples analyzed by MC (y-axis) *vs.* SFC (x-axis) **(A, C)** or downsampled SFC (x-axis) **(B, D)**. Major and minor cell populations are reported in panels **(A-D)**, respectively. Colors depict distinct populations whereas distinct symbols represent the different donors. *Axl DC, Axl^+^ dendritic cell; cMo, classical monocyte; HPC, hematopoietic precursor cell; iMo, intermediate monocytes; myDC, myeloid dendritic cell; M-MDSC, monocytic myeloid-derived suppressor cell; ncMo, non-classical monocyte; pDC, plasmacytoid dendritic cell; preDC, CD100^+^ dendritic cell precursor*.

Overall, similar expression patterns were observed in both platforms, allowing for the identification of the same populations of PBMC (n=24). These included the major lymphocyte populations of T cells, B cells and NK cells, granulocytes (basophils, left-over neutrophils) and minor IMC subsets of e.g., monocytes and DC (e.g., non-classical monocyte - ncMo - subsets, CD141^+^ myDC), including recently described populations (e.g., Axl^+^ DC) (**Figure 1** and **Supplementary Figures 1**-**4**). Additionally, a minor population of unassigned events was also detected in both approaches at similar levels, corresponding to cells for which the marker combination employed did not allow for reliable classification (e.g. innate lymphoid cells, CD56^bright^ NK cells). In addition, a good overall correlation (correlation coefficient = 0.99) was observed on the relative distribution of these cell populations between SFC and MC, independently of down sampling (**Figures 2A, B**) and even when only cell populations present at frequencies of <25% were considered (Pearson’s ρ = 0.97) (**Supplementary Figure 5**). However, when minor monocytic and DC subpopulations, typically identified based on different levels of expression (*vs.* absence/presence) of antigens, were evaluated (**Supplementary Figure 4**), non-down sampled SFC samples displayed a lower correlation with MC *vs.* the dSFC ones (**Figures 2C**, **D**; ρ = 0.90 *vs* 0.96, respectively). Similarly, when we evaluated the correlation between the two technologies for individual cell populations (**Supplementary Table 3**), lower correlation values (ρ ≤ 0.80) were observed for 7/30 (23%) and 3/30 (10%) individual populations in both non-downsampled and downsampled SFC *vs.* MC samples, respectively. Of note, good reproducibility (ρ > 0.80) between dSFC and MC was observed for the majority of the cell populations evaluated (27/30; 90%), including those present at very low frequencies (<0.01% of PBMC), except for neutrophils, monocytic myeloid-derived suppressor cells (M-MDSC) and NK cells. Furthermore, good correlation was also observed for individual donors (median and range Pearson’s ρ of 0.998 (0.986-0.999) and 0.998 (0.986-0.999) in MC vs. SFC and dSFC, respectively].

### Staining resolution of SFC *vs.* MC

A key factor for accurate and reproducible identification of the individual PBMC populations relies on the resolution of the identification markers, particularly in the case of IMC, which require discrimination according to the differential expression of shared markers (27). To determine potential differences in staining quality and resolution between the two technologies, we used the AOF between negative and positive reference populations (NRP and PRP, respectively) for each of the 21 individual markers used in both SFC and MC. AOF is a semi-quantitative metric based on the bimodal distribution of markers, which uses a range of 0-100%, where 0% indicates no overlap between two cell populations, 100% indicates full overlap, and AOF values ≥15% are reported to be associated with potential resolution issues (12, 49).

**Figure 3:**
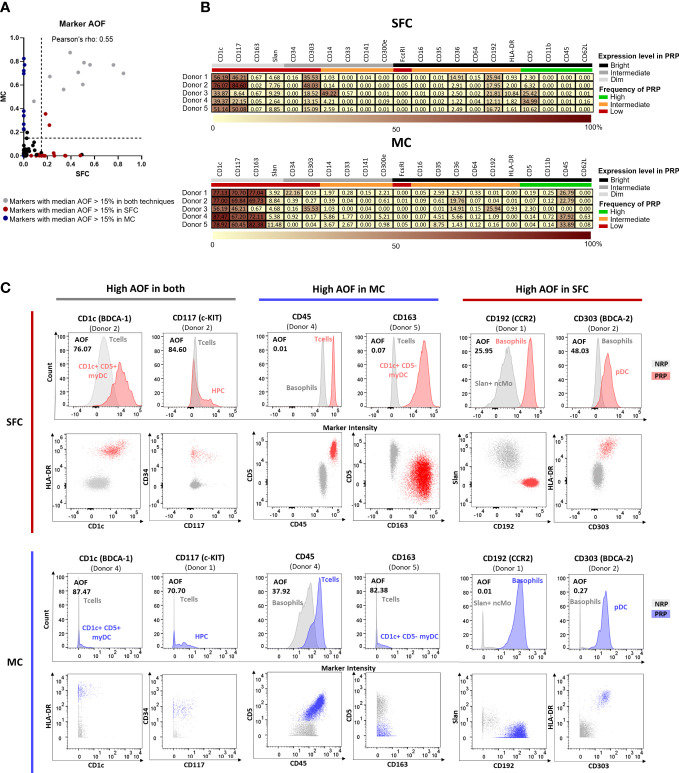
Average Overlap Frequencies (AOF) observed for markers measured by both spectral flow cytometry (SFC) and mass cytometry (MC). Correlation between AOF values obtained by SFC and MC is depicted in panel **(A)**. Markers with a median AOF > 15% are indicated in grey (both techniques), red (SFC) and blue (MC), respectively. Black dots indicate a median AOF ≤ 15% for both methods. In panel **(B)**, heatmaps show AOF values of SFC and MC for each marker and donor. Low AOF values are indicated in light yellow, whereas high values are shown in dark red. Information on the expression pattern of each marker (black, bright expression; grey, intermediate expression; light grey, dim expression) and frequency of positive reference population (PRP) is indicated (green, high frequency; orange, intermediate frequency; red, low frequency). Panel **(C)** depicts histograms and dot plots corresponding to those markers that showed a median AOF ≥15% (for each marker, the donor depicting the highest AOF is shown). Grey, negative reference population; red, positive reference population by SFC; blue, positive reference population by MC. *HPC, hematopoietic precursor cell; MC, mass cytometry; myDC, myeloid dendritic cells; ncMo, non-classical monocytes; NRP, negative reference population; pDC, plasmacytoid dendritic cell; PRP, positive reference population; SFC, spectral flow cytometry*.

Overall, a significant but marginal correlation of AOF between SFC and MC was observed (Pearson’s ρ = 0.55, p<0.0001) (**Figure 3A**), with low (<15%) median AOF values detected for 14/21 (67%) markers evaluated with both techniques (**Figure 3B**). In contrast, high AOF values were systematically observed in both SFC and MC for 2/21 (9.5%) markers which are dimly expressed on rare cell populations (CD1c and CD117) with median AOF of 51.1% and 46.2% for SFC and 77.1% and 67.7% for MC, respectively) (**Figures 3B, C**). Interestingly, for 4/21 (19%) markers systematic discrepancies between the two technologies were observed, despite not reaching statistical significance. Thus, CD163 and CD45 displayed higher median AOF values by MC (72.1% and 26.8%, respectively) *vs.* SFC (0.07% and 0.01%, respectively), whereas CD192 and CD303 displayed higher (>15%) median AOF values by SFC (18.0% and 18.5%, respectively) *vs.* MC (0.2% for both markers) (**Figures 3B, C**).

#### Intra-measurement variability

As myeloid subsets can be present at very low frequencies in blood, and thereby also PBMC (e.g., 0.001% of all PBMC) (**Supplementary Table 3**), high numbers of cells need to be measured to allow for their reliable identification of the populations (e.g., ≥1x10^6^ cells are required to identify a cluster of 10 cells present at a frequency of 0.001%). Ultimately this leads to longer acquisition times, particularly in the case of using MC (*vs.* SFC) due to the lower event rate used during cell measurements.

**Figure 4:**
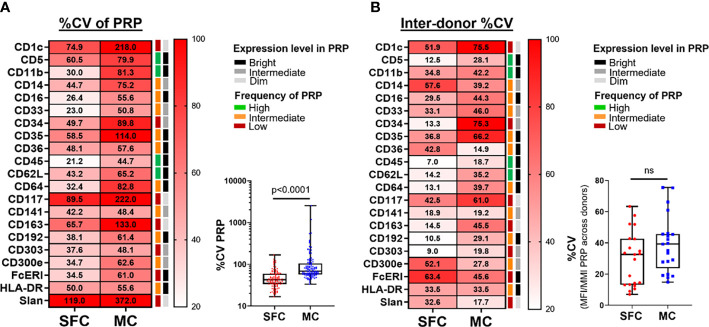
Coefficient of variation (%CV) during and between measurements made by spectral flow cytometry (SFC) *vs.* mass cytometry (MC). Panel **(A)** depicts the %CV for each marker (n=21) for its positive reference population (PRP). Median %CV is reported in the heatmap, whereas the overview of individual %CV for each marker in its PRP per donor is shown in the box plot. Panel **(B)** depicts the %CV of the median fluorescence intensity (MFI)/metal intensity (MMI) of a PRP across the different donors for SFC *vs.* MC. %CV of individual markers is reported in the heatmap, whereas the overall profile is shown in the box plot. Marker expression and frequency of the PRP are indicated by color (black, grey and light grey correspond to bright, intermediate and dim expression levels, respectively; green, orange and red reflect high, intermediate and low frequency of the PRP among all PBMC, respectively). Statistical differences were evaluated employing the Wilcoxon matched-pairs signed rank test. *CV, coefficient of variation; MC, mass cytometry; MFI, median fluorescence intensity; MMI, median metal intensity; PRP, positive reference population; SFC, spectral flow cytometry*. ns, not statistically significant.

Once cell populations are identified based on markers that show a continuous expression pattern (such as monocytic cell subsets, defined on different levels of expression of CD14, CD16, CD36, CD62L, FcεRI and CD62L) (**Supplementary Figures 2**, **3**), a high intra-sample variability due to e.g., instrument instability during longer data acquisition times in the cytometer, might be expected. This instability has a deleterious impact on data reproducibility, associated with greater heterogeneity of the marker expression pattern, reflected by higher %CV values. Therefore, we evaluated the %CV obtained for each marker in its corresponding PRP (**Supplementary Table 2**). Of note, %CV values obtained with MC were significantly higher for all evaluated markers (21/21; 100%) than those of SFC, with a median %CV of 68.0% *vs.* 42.5%, respectively (p <0.0001) (**Figure 4A**).

To gain insight into the causes of such higher heterogeneity observed for the MC *vs*. SFC staining patterns, we further investigated whether such differences harmed the identification and quantification of different populations, and whether or not they related to the (very) different acquisition times in our experiments with SFC *vs.* MC: median (range) of 15.9 min (9.6 – 16.9 min) *vs.* 159 min (122 – 276 min) for the acquisition of 1.2x10^6^-3.3x10^6^ (live) PBMC by SFC *vs.* MC, respectively. For this purpose, we determined the relative frequency of the different cell populations over time (during data acquisition in the cytometer) by dividing the total acquisition into 5 blocks of equal duration in the “Time” parameter. To avoid the potential bias of having different numbers of events in each time block for SFC and MC data files, dSFC files were used for this comparison. Of note, neutrophils and DC precursors (preDC) were excluded from this analysis, as <10 cells were detected in each time block for each of these two cell populations (**Supplementary Figure 6**).

Overall, a significantly lower number of cells was measured in the last time slot of each file for SFC (p=0.02). Despite this, the variation for most cell populations (15/22; 68.2%) was systematically observed within ±20% (median difference *vs.* the relative frequency reported in the first time slot), across all time points evaluated (**Supplementary Figure 6**). Systematic significant variations (absolute median deviation >20% *vs.* the first time block) were mainly detected in the last stage of acquisition (time frame 5) and were restricted to 3/22 (13.6%) cell populations evaluated by SFC (CD62L^-^ FcεRI^+^ cMo, iMo and CD1c^+^ CD14^low^ myDC) and 2/22 (9.1%) cell populations (CD36^-^ Slan^-^ ncMo and M-MDSC) measured by MC.

#### Reproducibility of SFC *vs.* MC measurement across different days

The reproducibility of data measured on different days is critical for the accurate interpretation of data in both research and clinical settings. Approaches to minimize the impact of samples collected at different timepoints exist (e.g., batch correction methods or storage of samples for delayed measurements on the same day), but these cannot be applied for fast reporting of results. To assess the reproducibility of measurements performed on different days, we evaluated the variability of the staining patterns obtained across all samples. As the same donors were evaluated with both technologies, we would expect a similar dispersion across individuals in both techniques (i.e., due to the biological variation). Consequently, differences observed between the two technological platforms are most likely due to technical variability. To address this we evaluated the %CV of the median fluorescence intensity (MFI)/median metal intensity (MMI) of each marker in its corresponding PRP across all donors (**Figure 4B** and **Supplementary Table 2**). Overall, a higher variability between donors was observed for MC *vs.* SFC: 14/21 (66.7%) markers showed higher %CV with MC *vs.* only 5/21 (23.8%) markers within SFC (p=0.01), with an overall median (range) %CV of 39.2% (14.9% - 75.5%) *vs.* 32.6% (7.0% - 63.4%), respectively (p>0.05).

## Discussion

Currently, SFC and MC are the preferred technologies for immunophenotypic profiling of immune cells with high numbers of markers (>20), allowing for immune monitoring in clinical settings. In recent years, several groups have compared the performance of the two platforms (12, 18, 19). In these comparisons, frozen samples were used, batch analysis was performed and/or limited focus was on IMC. Analysis of IMC is particularly challenging, due to (frequent) lack of lineage-specific proteins and because multiple myeloid populations are defined *via* different levels of expression (rather than the presence *vs.* absence) of one or more markers, which are co-expressed in distinct IMC populations. Furthermore, immune monitoring for diagnostic patient care requires fast response times and measuring fresh samples over consecutive days. This impedes the application of strategies aimed at minimizing technical variation, such as batch correction or barcoding. Here we compared for the first time the performance of SFC *vs.* MC for the identification, characterization and enumeration of circulating IMC, aimed at potential implementation in clinical laboratories.

In this study, we employed two 24-marker SFC and 33-marker MC panels which allowed for the evaluation of 21 common parameters. Therefore, both SFC and MC combinations included parameters that were not employed in this study. This is mostly due to the fact that the MC panel was designed for investigation of IMC populations across five different tissues (i.e., bone marrow, PB, skin, colon and peritoneal dialysate), and additional antibodies were added to allow the study of myeloid populations in all the tissues. These included markers for exclusion of lymphoid populations (e.g., CD3, CD19) and identification of subsets not present in PB (e.g., CD11c, CD1a, CD207 for CD1a+ myDC and Langerhans cells in skin; CD206 for M2-polarized macrophages; CD123 for basophil and pDC precursors in bone marrow). Furthermore, for some markers redundancy was evaluated in PB, but not in the other tissues and, therefore, needed to be present for those tissues not addressed in the present study. Examples of the latter include CD100 for preDC (identified in PB based on CD34 and HLA-DR expression), Axl for Axl^+^ DC (which in PB show a unique CD303, CD33, CD5 and HLA-DR profile) and CLEC9A for CD141^+^ myDC (CD141 is less specific than CLEC9A in the other tissues evaluated) (27).

Overall, MC was associated with a significantly lower (half) recovery of (live) cells *vs.* SFC. To the best of our knowledge, this is the first time these differences are reported, mainly because in previous studies, fixed numbers of cells were measured per sample, whereas all the stained sample was analyzed in our study (12, 19). The lower cell recovery by MC *vs.* SFC is likely caused by the longer sample preparation and staining protocols with more centrifugation steps, which lead to the loss of up to 50% of cells (50). In addition, a limited instrument-related cell recovery has also been reported (30% to 50%) for MC (17, 50).

Despite the significant loss of cells, still all 24 different immune cell populations (including 21 IMC populations), identified with the common 21-marker combination used, could be unequivocally identified and quantified in both the SFC and MC instruments, allowing us to compare the performance of the two platforms for the identification and enumeration of a significantly larger set of myeloid cell populations than evaluated in previous reports, which were restricted to ≤8 cell populations (12, 46). Furthermore, these studies typically focus on relatively large populations, defined based on clear bimodal expression of markers, ultimately reducing the power of the comparisons. In order to address these limitations here we aimed at an in-depth dissection of the IMC compartment in PB mononuclear cells, including minor, recently described populations (e.g. Axl+ dendritic cells), as well as subsets defined based on the level of expression of the markers (e.g. cMo, ncMo and myDC CD1c+ subpopulations).

In line with previous studies, here we observed a very good correlation between SFC and MC for all major cell subsets evaluated, with correlation coefficients of 0.99 *vs.* 0.98 (12) even though our panel lacked traditional lineage markers such as CD3 (Tcells), CD19 (B cells) and CD56 (NK cells), and light scatter information was not included for identification of the cell populations in the SFC data files. Likewise, the high correlation was also observed for individual donors (Pearson’s ρ systematically >0.98). More importantly, we reported a major improvement in the correlation between the two techniques for the study of the myeloid cell compartment compared to previous studies [Pearson’s ρ of 0.90 *vs.* 0.27 (12)]. Similarly, a good correlation (Pearson’s ρ>0.8) was observed for 90% of the individual populations evaluated. This includes very infrequent (<0.1% of PBMC) cell subsets, defined *via* a limited number of parameters, which showed lower reproducibility in earlier reports (27), such as preDC, hematopoietic precursor cells (HPC), CD141^+^ myDC, Axl^+^ DC and CD36^+^ Slan^+^ ncMo. The higher correlation in our study is most likely due to the evaluation of higher cell numbers (>12x10^5^ *vs.* 0.5-2.5x10^5^, in other studies) (12, 19, 46), together with a more comprehensive design of the IMC antibody panels. Furthermore, the different numbers of cells evaluated with the two technological platforms (18) could also contribute to the poorer correlation observed in previous studies, since a clear improvement was observed in our study when SFC data was scaled down to match the number of PBMC evaluated by MC. Of note, for three cell populations, still low correlations were observed between the two approaches, even when dSFC (instead of SFC) samples were used for comparison with MC. This is probably due to their low frequency (<0.005% for neutrophils with <50 cells/sample), the lack of optimal marker combinations for their identification (e.g., NK cells) and/or the combination of both (e.g., M-MDSC).

Regarding marker resolution, AOF values revealed an overall good resolution for two-thirds of the markers with both technological platforms, in line with previous reports (0-87% overlap) (12). However, a low resolution was observed with both SFC and MC for two markers (CD1c and CD117). This is likely due to i) the low frequency of their PRP, together with ii) their low expression levels and/or iii) the heterogeneous expression profile on their PRP (e.g., CD117) (51). Despite the high resolution observed for most markers by both SFC and MC, there was an overall lower correlation between the two techniques when compared to other reports (12). This was due, at least in part to discrepancies between the two approaches for some markers (i.e., CD45 and CD163 showed lower resolution in MC, while CD192 and CD303 were associated with higher AOF with SFC). Overall, such discrepancies, particularly for those four markers, might be related to i) the use of different antibody clones in the two panels (e.g., for CD192 and CD303); ii) the impact of the spread of the signal in case of SFC (52), and iii) the relative brightness of the label selected, e.g., CD45 and CD163 were conjugated with 89Y and 141Pr in the MC panel, which are heavy metals that perform outside the highest sensitivity range of MC (isotopes 153-176) (13), resulting in suboptimal signal intensities. Alternatively, a combination of these three can also occur since e.g., different clones, conjugated with a fluorochrome of intermediate intensity (PerCP eFluor710), affected by spread coming from the CD11b BV711 and CD33 PE Cy7 markers which are strongly expressed on the NRP used to calculate the resolution for CD303 (basophils). Another reason for the discrepancy could be the presence of reactive/aberrant phenotypes, detected with different sensitivity with the two platforms. However, the set of markers employed for the comparison was selected based on previously developed and validated combinations (27) (patent “Means and methods for multiparameter cytometry-based leukocyte subsetting”; PCT/NL2020/050688, filing date 5 November 2019). Furthermore, it has been previously (successfully) employed for the study of IMC in several models where reactive/aberrant phenotypes can be detected (e.g., infection, vaccination, hemato-oncology), as well as a backbone for functional studies employing *in vitro* activation (32–36). Overall, this highlights the power of the combination of markers employed in the study even in cases where “reactive”/aberrant phenotypes are present. However, as the above-mentioned combination was developed and validated for flow cytometry, it is possible that, the detection of dim expression for those markers with lower resolution in MC *vs.* SFC (i.e., CD45 and CD163) could potentially be impaired in the former. Nevertheless, in this study no aberrant phenotypes were detected by SFC and, therefore, this potential reduction in sensitivity for detection of dimly expressed CD45 and/or CD163 would not have an impact in the results.

The possibility to evaluate the information generated by both approaches employing the same scale would allow for a clearer visualization and interpretation of the data. However, since the scale of the data is drastically different in MC vs. SFC (10^4^ *vs*. 10^6^ range, respectively), its transformation would potentially affect the interpretation of the performance of the platforms. In line with this, the generation of artifacts has been previously reported for batch correction methods applied to MC data, e.g. by leading to differences in the numbers of zero-valued events (either inflating the zeros to non-zero values introducing noise, or compressing the lowest non-zero events to zero, resulting in loss of information) (53). Therefore, in this study, no transformation of the data was performed to avoid any bias and provide a fair comparison of the technologies.

For highly-reproducible analysis of (minor) IMC populations, longer acquisition times were required in our study to evaluate a minimum number of cells, which ranged from a median of 16 min for SFC to 159 min for MC. Because of such differences in the duration of data acquisition, we evaluated the impact of the speed of acquisition on the staining patterns observed for PRP, and its potential impact on their identification and quantification. Overall, MC was associated with a higher %CV for all markers investigated, regardless of the frequency and brightness of the maker on the PRP. These findings would point to the existence of a direct association between longer acquisition times required for MC and more variable staining patterns. Although approaches exist that might be implemented to limit intra-measurement variability in MC, such as signal normalization during the measurement using EQ beads, the longer acquisition times might also result in a loss of detector sensitivity or changes in the efficiency of plasma ionization (54). Despite the increased variability observed with MC, no significant variation in time (i.e., absolute difference in population frequency <20% *vs.* the first time slot during measurement) was observed for most cell populations in both SFC and MC. Interestingly, most deviations were observed in the very last stage of acquisition, particularly for SFC samples, as a result of a significantly lower number of cells evaluated with this technology in the last time slot during acquisition. Such decreased frequency of cells measured in the later acquisition periods contrasts with the stable measurement in terms of the number of cells observed with MC. This difference is most likely the result of a dilution of the sample performed in the last stage of measurement to maximize recovery while avoiding introducing air in the system, for the samples evaluated with SFC. Additionally, this effect is minimized in MC, since cells are continuously being gently mixed and cooled during data acquisition in the cytometer.

The use of SFC and MC measurements in clinical settings typically requires a fast turnaround of results. Because of this, multiplexing of samples or freezing until collection is complete, followed by measurement of all samples collectively, are not feasible in diagnostic laboratories. Instead, samples need to be evaluated as soon as possible after collection. Overall, this results in potential day-to-day variations due to daily differences in the instruments. To determine daily variation levels between SFC and MC, we measured paired samples which are expected to show the same biological variability between them, any deviations thereby, being most likely due to technical variability. Overall, higher inter-donor variability was observed for MC *vs.* SFC. The lower variability of SFC might be due to the daily instrument quality control performed, where detector gains are daily adjusted so that the MFI of standard calibrator beads used in this procedure reach the same target MFI value every day (55). However, further studies in which the same sample is measured over different days in both instruments would allow for a more accurate overview of the absolute (*vs.* relative to each other) degree of technical variability associated with each technological platform, and thereby, derive definitive conclusions about daily technical variation. Despite this, our results indicate that, compared to MC, SFC might be a more robust technology for situations where samples need to be processed and analyzed immediately after collection. However, the use of additional strategies aimed at reducing the higher daily technical variability observed with MC, such as the use of batch correction methods or sample barcoding/multiplexing needs to be evaluated (29, 30, 50, 56).

Of note, while we specifically aimed at the study of the myeloid compartment, the potential of both SFC (in research instruments with 5 laser lines) and MC could be further expanded to >40 markers. This would allow for the inclusion of additional markers for further lymphoid subsetting, ultimately increasing the power of the results (higher number of populations and markers evaluated).

In summary, in line with previous reports, our results suggest that SFC and MC yield highly comparable results for monitoring immune cells (12, 46). However, while both technologies can be employed for these studies, whenever careful panel design is performed, the selection of a platform for future applications should also take into account other differential features of both technologies (**Table 1**). Spectral overlap and spread are not an issue with MC and, thereby, panel design for populations with a high number of co-expressed markers can be more straightforward than in fluorescence-based SFC, while the latter measurements are simpler and faster, allowing for quicker analysis and shorter turnaround times to results.

**Table 1:** Performance of spectral flow cytometry (SFC) *vs.* mass cytometry (MC) for the study of innate myeloid cells.

FEATURE	SFC	MC
*Panel design (>20 markers) for IMC*	Complex	Simple
*Cell size/complexity measurement*	Yes	No
*Autofluorescence*	Yes	No
*Single stained controls required*	Yes	Advised
*Measurement throughput*	≈ 6 min/10^6^ cells	≈ 113 min/10^6^ cells
*Recovery (median)*	≈ 53.1%	≈ 26.8%
*Staining resolution* *(% markers with median AOF<15%)*	Good (81%; 17/21 markers)	Good (81%; 17/21 markers)
*Intra-measurement variability* *(% markers with higher median %CV PRP; median %CV)*	Lower (0%; 0/21 markers; %CV 42.5%)	Higher (100%; 21/21 markers; %CV 68.0%)
*Reproducibility across different days* *(% markers with higher inter-donor variability)*	Higher (23.8%; 5/21 markers)	Lower (66.7%; 14/21)

AOF, Average Overlap Frequency; CV; coefficient of variation; IMC, innate myeloid cells; MC, mass cytometry; PRP, positive reference population; SFC, spectral flow cytometry.

## Author contributions

CT, PD and JJMvD designed the study and performed the project administration. KvdP, AdJ, AL, SK, BN, and IdL processed the samples. SC and CT designed and optimized the SFC panel. MH and KvdP designed the MC panel. PD and KvdP performed the conjugation and titration of antibodies for MC. KvdP, MH and PD measured the MC samples. KvdP, CT and AdJ measured the SFC samples. KvdP and CT performed the pre-processing of the SFC and MC files. KvdP analyzed the MC and SFC samples. KvdP, CT and IK made the figures and performed the statistical analyses. KvdP, CT, PD, AO and JJMvD wrote the manuscript. All authors contributed to the article and approved the submitted version.
