# Supplementary material for: Performance of spectral flow cytometry and mass cytometry for the study of innate myeloid cell populations
Source: Front Immunol. 2023 May 19;14:1191992. doi: 10.3389/fimmu.2023.1191992 (PMC10235610; doi:10.3389/fimmu.2023.1191992)
Supplement: Supplementary file 1 [file DataSheet_1.docx]

# Performance of spectral flow cytometry and mass cytometry for the study of innate myeloid cell populations

Kyra van der Pan^1^, Indu Khatri^1^, Anniek L de Jager^1^, Alesha Louis^1^, Sara Kassem^1^, Brigitta AE Naber^1^, Inge F de Laat^1^, Marjolijn Hameetman^2^, Suzanne ET Comans^1^, Alberto Orfao^3^, Jacques JM van Dongen^1,3^*, Paula Díez^1,4†^, Cristina Teodosio^1,3†^, on behalf of the EuroFlow and TiMaScan consortia

^1^Department of Immunology, Leiden University Medical Center (LUMC), Leiden, The Netherlands

^2^Flow Cytometry Core Facility, Leiden University Medical Center (LUMC), Leiden, The Netherlands

^3^Translational and Clinical Research Program, Cancer Research Center (IBMCC; University of Salamanca - CSIC); Cytometry Service, NUCLEUS; Department of Medicine, University of Salamanca and Institute of Biomedical Research of Salamanca (IBSAL), Salamanca, Spain

^4^ Health Research Institute of Asturias (ISPA) and Asturias Central University Hospital (HUCA), Oviedo, Asturias, Spain

† These authors contributed equally to this work and share last authorship

*** Correspondence:** Prof. J.J.M van Dongen, MD, PhD

Leiden University Medical Center (LUMC)

[J.J.M.van_Dongen@lumc.nl](mailto:J.J.M.van_Dongen@lumc.nl)

Cristina Teodosio, PhD

University of Salamanca, Spain

[cristina.teodosio@usal.es](mailto:cristina.teodosio@usal.es)

**Running title** (max. 5 words)**:** Spectral vs. mass-cytometry for myeloid-cells

**Keywords:** mass cytometry, CyTOF, spectral flow cytometry, myeloid cells, immunophenotyping

## **Supplementary Material and Methods**

### **Antibody conjugation for mass cytometry**

Carrier-, glycerol- and BSA-free purified IgG antibodies and the MaxPar® X8 Antibody Labeling Kit were used for antibody conjugation for mass cytometry, according to the manufacturer’s instructions (Standard Biotools, San Francisco, CA). Briefly, MaxPar® polymers were thawed, resuspended in 95 µL of L-buffer (MaxPar® X8 Antibody Labeling Kit) and incubated at 37 °C for 40 min in the presence of 5 µL of the heavy-metal tag. The polymer-metal solution was transferred into a 3 kDa Amicon Ultra 500 µL filter (Sigma-Aldrich, Saint Louis, MO), and washed with 200 µL L-buffer by centrifuging at 12,000 g for 25 min at room temperature (RT), followed by another wash with 400 µL C-buffer (MaxPar® X8 Antibody Labeling Kit) at 12,000 g for 30 min (RT). In parallel, 100 µg of each IgG-purified antibody was prepared by washing with R-buffer (MaxPar® X8 Antibody Labeling Kit; total volume 400 µL) followed by centrifugation at 12,000 g for 10 min at RT in a 50 kDa Amicon Ultra 500 µL filter (Sigma-Aldrich). Flowthrough was discarded and antibodies were reduced by adding 100 µL 0.5M Bond-Breaker^TM^ TCEP solution (Thermo Fisher, Waltham, MA) followed by incubation at 37 °C for 30 min. Whenever available, purified antibodies were purchased as MaxPar® Ready, and therefore did not require the pre-processing steps prior to treatment with the TCEP solution. Antibodies were washed twice with C-buffer (total volume 400 µL) and centrifuged at 12,000 g for 10 min (RT). Polymers and antibodies were incubated in a total of 100 µL of C-buffer in the 50 kDa filter at 37 °C for 90 min (RT). Polymer-antibody solutions were then washed three times in 400 µL of W-buffer (MaxPar® X8 Antibody Labeling Kit) by centrifuging at 12,000 g for 10 min (RT). Labelled antibodies were recovered by adding 50 µL of Antibody PBS Stabilizer (CANDOR Bioscience GmbH, Wangen im Allgäu, Germany) before inverting the 50 kDa filter over a new tube and centrifuging at 1,000 g for 2 min. This process was repeated once, and an additional 100 µL Antibody PBS Stabilizer was added (for a total volume 200 µL) before the conjugated antibodies were stored at 4 °C.

The conjugation of each antibody was tested employing either MACS® Comp Beads (Miltenyi Biotec, Bergisch Gladbach, Germany) or OneComp eBeads™ Compensation Beads (Thermo Fisher), depending on the nature of the antibody (recombinant or not), according to the manufacturer’s guidelines. Briefly, one drop of OneComp eBeads™ Compensation Beads (Thermo Fisher) was incubated with 2 µL of conjugated antibody and 100 µL Cell Staining Buffer (CSB; Standard Biotools, San Francisco, CA) for 30-45min (RT). Due to the nature of the Slan-custom pure antibody (recombinant antibody), its conjugation was tested with MACS® Comp Beads (Miltenyi Biotec). Briefly, the beads were washed with 2 mL of CSB, centrifuged at 500 g for 5 min, pooled and washed for an additional three times with CSB (500 g at 5 min). Pooled beads were left pelleted at 4 °C until acquisition; then, they were resuspended in MilliQ and acquired on the Helios mass cytometer (Standard Biotools). When different batches of conjugated antibodies were required, intensity differences were compared between sets to ensure proper staining and comparability between batches.

## **Figure legends**

**Supplementary Figure 1: Uniform Manifold Approximation and Projection (UMAP) representation of paired peripheral blood mononuclear cell (PBMC) samples analysed by spectral flow cytometry (SFC) (downsampled file – dSFC -) and mass cytometry (MC), based on a common combination of 21 markers.** Major cell populations are depicted in the top rows (panels **A-F**), whereas an in-depth analysis of the myeloid cell compartment (excluding basophils) plus hematopoietic precursor cells is depicted in the bottom rows (panels **G-J).** Overview of the relative intensity of each of the markers employed in the comparative analysis is reported in panels **B, D, H** and **J,** overlayed on the corresponding UMAP.

**Supplementary Figure 2:** **Representative overview of phenotypical patterns of identified leukocyte populations in spectral flow cytometry files.** The phenotypical patterns of granulocytes (panel **A, B**), lymphocytes (panel **C**), precursor cells (panel **D**), dendritic cells (panels **E** and **F**) and monocytes and M-MDSC(panel **G**) in spectral flow cytometry were shown. This gating strategy is only meant to provide insight in phenotypical patterns, as the analyses in this publication were performed unbiasedly and therefore no manual gating was performed.

*Axl DC, Axl^+^ dendritic cell; cMo, classical monocytes; HPC, hematopoietic precursor cells; iMo, intermediate monocytes; myDC, myeloid dendritic cells; M-MDSC, monocytic myeloid-derived suppressor cells; ncMo, non-classical monocytes; pDC, plasmacytoid dendritic cells; preDC, dendritic cell precursors.*

**Supplementary Figure 3:** **Representative overview of phenotypical patterns of identified leukocyte populations in mass cytometry files.** The phenotypical patterns of granulocytes (panel **A, B**), lymphocytes (panel **C**), precursor cells (panel **D**), dendritic cells (panels **E** and **F**) and monocytes and M-MDSC (panel **G**) in mass cytometry were shown. This gating strategy is only meant to provide insight in phenotypical patterns, as the analyses in this publication were performed unbiasedly and therefore no manual gating was performed.

*Axl DC, Axl^+^ dendritic cell; cMo, classical monocytes; HPC, hematopoietic precursor cells; iMo, intermediate monocytes; myDC, myeloid dendritic cells; M-MDSC, monocytic myeloid-derived suppressor cells; ncMo, non-classical monocytes; pDC, plasmacytoid dendritic cells; preDC, dendritic cell precursors.*

**Supplementary Figure 4: Overview of staining patterns obtained for different populations of peripheral blood mononuclear cells (PBMC) by spectral flow cytometry (SFC) and mass cytometry (MC) in a representative donor (donor 3).** Bivariate plots depicting the immunophenotypic profile observed in samples evaluated by SFC, including also the downsampled files (dSFC) and MC. Major cell populations (B, T and NK cells and basophils) are shown in panels **A-D**. Hematopoietic precursor cells (HPC) are depicted in panel **E**, whereas subsetting of dendritic cell populations is exhibited in panel **F**. Expression patterns for identification of major (panel **G**) and minor (panels **H** and **I**) subsets of monocytes are also displayed. Information on the immunophenotypic profile used for the identification of the distinct cell populations is shown between brackets. Panels **A-D** depict pseudocolour plots, whereas in panels **E-I** distinct subsets are indicated in different colors.

*Axl DC, Axl^+^ dendritic cell; cMo, classical monocyte; dSFC, downsampled spectral flow cytometry; MC, mass cytometry; iMo, intermediate monocyte; myDC, myeloid dendritic cell; ncMo, non-classical monocyte; pDC, plasmacytoid dendritic cell; SFC, spectral flow cytometry.*

**Supplementary Figure 5: Correlation between the frequency of different populations of peripheral blood mononuclear cells (PBMC) measured by spectral flow cytometry (SFC) *vs.* mass cytometry (MC).** Correlation between the percentage of different populations of PBMC (present at frequencies <25%) identified in paired samples analysed by MC (y-axis) *vs.* spectral flow cytometry (x-axis) (panel **A)** or downsampled SFC (x-axis) (panel **B**). Colours depict distinct populations whereas different symbols represent the distinct donors.

*Axl DC, Axl^+^ dendritic cell; cMo, classical monocyte; HPC, hematopoietic precursor cell; iMo, intermediate monocyte; myDC, myeloid dendritic cell; M-MDSC, monocytic myeloid-derived suppressor cell; ncMo, non-classical monocyte; pDC, plasmacytoid dendritic cell; preDC, CD100^+^ dendritic cell precursor.*

**Supplementary Figure 6:** **Intra-measurement variability assessed as the impact on the percentage of different cell populations over time, during data acquisition in the mass cytometry (MC) and spectral flow cytometry (SFC) instruments.** The total number of leukocytes per identical time frame is depicted in panel **A**. Panels **B-W** show differences in the frequencies of the distinct cell populations observed over time, normalized to the first time frame, for downscaled spectral flow cytometry (dSFC) and MC paired samples. Major lymphoid populations (panels **B-D**), basophils (panel **E**), monocyte subsets (panels **F-N**), dendritic cell populations (panels **O-T**), hematopoietic precursor cells (panel **U**), monocytic myeloid-derived suppressor cells (M-MDSC) (panel **V**) and unclassified PBMC (panel **W**) are shown. Data is reported as median and 95% confidence interval, and it is depicted in different shades of grey. Statistically significant differences were determined by the Kruskal-Wallis and the post hoc Dunn's multiple comparisons tests (* p-value <0.05; ** p-value <0.01; *** p-value <0.001; **** p-value <0.0001).

*Axl DC, Axl^+^ dendritic cell; cMo, classical monocytes; DC, dendritic cells; HPC, hematopoietic precursor cells; iMo, intermediate monocytes; dSFC, downsampled spectral flow cytometry data files; MC, mass cytometry; myDC, myeloid dendritic cells; M-MDSC, monocytic myeloid-derived suppressor cells; ncMo, non-classical monocytes; pDC, plasmacytoid dendritic cells; n.s., not statistically significant. Note: data on neutrophils and dendritic cell precursors (preDC) is not shown due to the low number of cells evaluated at each timepoint for individual samples (<10 cells per time frame).*

**Supplementary Table 1: Heavy-metal and fluorochrome-conjugated antibodies (Ab) and reagents employed for the study of innate myeloid cells by mass cytometry (MC) and spectral flow cytometry (SFC), respectively.**

|  | **Marker** | **Platform** | **Label** | **Ab Clone** | **Reagent identifier** |
| --- | --- | --- | --- | --- | --- |
| **Common to SFC and MC panels** | **CD1c** (BDCA-1) | **MC** | 166 Er‡,† | L161 | BioLegend Cat# 331502, RRID:AB_1088995 |
|  |  | **SFC** | SB436 |  | Thermo Fisher Scientific Cat# 62-0015-42, RRID:AB_2762426 |
|  | **CD5** | **MC** | 175 Lu‡ | UCHT2 | BioLegend Cat# 300627, RRID:AB_2563756 |
|  |  | **SFC** | FITC |  | BioLegend Cat# 300606, RRID:AB_31409 |
|  | **CD11b** | **MC** | 174 Yb‡ | ICRF44 | BioLegend Cat# 301337, RRID:AB_2562811 |
|  |  | **SFC** | BV711 |  | BioLegend Cat# 301344, RRID:AB_2563792 |
|  | **CD14** | **MC** | 164 Dy‡ | M5E2 | BioLegend Cat# 301843, RRID:AB_2562813 |
|  |  | **SFC** | Qdot 800 | TüK4 | Thermo Fisher Scientific Cat# Q10064, RRID:AB_2556449 |
|  | **CD16** | **MC** | 155 Gd‡ | 3G8 | BioLegend Cat# 302051, RRID:AB_2562814 |
|  |  | **SFC** | AF700 |  | BD Biosciences Cat# 560713, RRID:AB_1727430 |
|  | **CD33** | **MC** | 142 Nd‡ | WM53 | BioLegend Cat# 303419, RRID:AB_2562818 |
|  |  | **SFC** | PE Cy7 | P67.6 | BD Biosciences Cat# 333952, RRID:AB_2713932 |
|  | **CD34** | **MC** | 167 Er‡ | 581 | BioLegend Cat# 343531, RRID:AB_2562837 |
|  |  | **SFC** | AF647 | 561 | BioLegend Cat# 343618, RRID:AB_2632632 |
|  | **CD35** | **MC** | 170 Er‡ | E11 | BioLegend Cat# 333402, RRID:AB_1089032 |
|  |  | **SFC** | BV605 |  | BD Biosciences Cat# 744276, RRID:AB_2742114 |
|  | **CD36** | **MC** | 156 Gd‡,† | CLB-IVC7 | N/A |
|  |  | **SFC** | PerCP Cy5.5 |  | Immunostep Cat# 36PP5.52, RRID:AB_2848146 |
|  | **CD45** | **MC** | 89 Y | HI30 | Fluidigm Cat# 3089003, RRID:AB_2661851 |
|  |  | **SFC** | AF532 |  | Thermo Fisher Scientific Cat# 58-0459-42, RRID:AB_11218673 |
|  | **CD62L** | **MC** | 161 Dy‡ | DREG-56 | BioLegend Cat# 304835, RRID:AB_2563758 |
|  |  | **SFC** | BV480 |  | BD Biosciences Cat# 566111, RRID:AB_2739513 |
|  | **CD64** | **MC** | 169 Tm‡ | 10.1 | BioLegend Cat# 305029, RRID:AB_2563759 |
|  |  | **SFC** | PE Cy5 |  | EXBIO Praha Cat# T8-644, RRID:AB_2891254 |
|  | **CD117** (c-KIT) | **MC** | 173 Yb‡ | 104D2 | BioLegend Cat# 313223, RRID:AB_2562829 |
|  |  | **SFC** | PE Dazzle594 |  | BioLegend Cat# 313226, RRID:AB_2566213 |
|  | **CD141** (BDCA-3) | **MC** | 147 Sm‡ | 1A4 | BD Biosciences Cat# 559780, RRID:AB_397321 |
|  |  | **SFC** | BB515 |  | BD Biosciences Cat# 565084, RRID:AB_2739058 |
|  | **CD163** | **MC** | 141 Pr‡ | GHI/61 | BioLegend Cat# 333602, RRID:AB_1088991 |
|  |  | **SFC** | BV650 |  | BD Biosciences Cat# 563888, RRID:AB_2738468 |
|  | **CD192** (CCR2) | **MC** | 162 Dy‡ | K036C2 | BioLegend Cat# 357202, RRID:AB_2561851 |
|  |  | **SFC** | BV786 | LS132.1D9 | BD Biosciences Cat# 747855, RRID:AB_2872317 |
|  | **CD300e** (IREM2) | **MC** | 146 Nd‡ | UP-H2 | Immunostep Cat# IREM2PU-01MG, RRID:AB_11141324 |
|  |  | **SFC** | APC |  | Immunostep Cat# IREM2A-100T, RRID:AB_11140615 |
|  | **CD303** (BDCA-2) | **MC** | 145 Nd‡ | AC144 | Miltenyi Biotec Cat# 130-108-063, RRID:AB_2661176 |
|  |  | **SFC** | PerCP-eFluor 710 | 201A | Thermo Fisher Scientific Cat# 46-9818-42, RRID:AB_11149131 |
|  | **FcεRI** | **MC** | 160 Gd‡ | AER-37 | BioLegend Cat# 334602, RRID:AB_1227649 |
|  |  | **SFC** | eFluor 450 |  | Thermo Fisher Scientific Cat# 48-5899-42, RRID:AB_2574088 |
|  | **HLA-DR** | **MC** | 149 Sm‡ | L243 | BioLegend Cat# 307651, RRID:AB_2562826 |
|  |  | **SFC** | BV570 |  | BioLegend Cat# 307638, RRID:AB_2650882 |
|  | **Slan** | **MC** | 144 Nd‡,† | REA1050 | N/A |
|  |  | **SFC** | PE | DD.1 | Miltenyi Biotec Cat# 130-119-867, RRID:AB_2784456 |
| **MC** | **CD1a*** | **MC** | 151 Eu‡ | HI149 | BioLegend Cat# 300102, RRID:AB_314016 |
|  | **CD2*** | **MC** | 163 Dy‡ | RPA-2.10 | BD Biosciences Cat# 555324, RRID:AB_395731 |
|  | **CD3*** | **MC** | 158 Gd‡ | UCHT1 | BioLegend Cat# 300443, RRID:AB_2562808 |
|  | **CD11c*** | **MC** | 143 Nd‡ | B-ly6 | BD Biosciences Cat# 555391, RRID:AB_395792 |
|  | **CD19*** | **MC** | 159 Tb‡ | HIB19 | BioLegend Cat# 302247, RRID:AB_2562815 |
|  | **CD56*** | **MC** | 153 Eu‡ | HCD56 | BioLegend Cat# 318345, RRID:AB_2562830 |
|  | **CD100*** | **MC** | 165 Ho‡ | A8 | BioLegend Cat# 328401, RRID:AB_1236386 |
|  | **CD123*** | **MC** | 152 Sm‡ | 6H6 | BioLegend Cat# 306027, RRID:AB_2562823 |
|  | **CD206*** | **MC** | 172 Yb‡ | 19.2 | BD Biosciences Cat# 555953, RRID:AB_396249 |
|  | **CD207*** | **MC** | 154 Sm‡ | 10E2 | BioLegend Cat# 352202, RRID:AB_10898115 |
|  | **Axl*** | **MC** | 168 Er‡ | 108724 | R & D Systems Cat# MAB154, RRID:AB_2062558 |
|  | **CLEC9a*** | **MC** | 148 Nd‡ | 683409 | R & D Systems Cat# MAB6049, RRID:AB_10889849 |
|  | **Intercalator-Rh**** | **MC** | 103Rh | N/A | Fluidigm Product nr. 201103A |
|  | **Intercalator-Ir**** | **MC** | 191Ir, 193Ir | N/A | Fluidigm Product nr. 201192A |
| **SFC** | **CD13*** | **SFC** | APC Cy7 | WM-15 | BioLegend Cat# 301710, RRID:AB_2289620 |
|  | **CD38*** | **SFC** | BV510 | HIT2 | BioLegend Cat# 303540, RRID:AB_2616792 |
|  | **CD133*** | **SFC** | BV421 | 293C3 | BD Biosciences Cat# 566598, RRID:AB_2739756 |
|  | **Cell viability**** | **SFC** | Zombie NIR | N/A | Biolegend Cat#423105 |

*, Not shared between SFC and MC panels and, therefore not used for the comparative analyses; **, The viability marker and DNA marker were used in the clean-up steps but not included in subsequent analyses; ‡, Conjugated in-house; †, Antibodies manufactured upon request to obtain glycerol- and carrier-free products and therefore do not have a catalogue number; N/A, not applicable.

***Abbreviations:*** *AF, Alexa Fluor; APC, allophycocyanin; BB, Brilliant Blue; BV, Brilliant Violet; Cy, cyanine; FITC, fluorescein isothiocyanate; Dy, dysprosium; Er, erbium; Eu, europium; Gd, gadolinium; Ir, iridium; Ho, holmium; Lu, lutetium; MC, mass cytometry; Nd, neodymium; PE; phycoerythrin; PerCP, Peridinin-Chlorophyll-Protein; Pr, praseodymium; Rh, rhodium; SFC, spectral flow cytometry; SB, Super Bright; Sm, samarium; Tb, terbium; Tm, thulium; Qdot, quantum dot; Y, yttrium; Yb, ytterbium.*

**Supplementary Table 2: Positive (PRP) and negative (NRP) reference cell populations used for the calculation of Average Overlap Frequencies (AOF) and intra- and inter-measurement variability assessment.**

| **Marker** | **PRP** | **NRP** |
| --- | --- | --- |
| **CD1c** | CD1c^+^ CD14^-^ CD5^+^ myDC | T cells |
| **CD5** | T cells | Basophils |
| **CD11b** | FcεRI^-^ cMo | pDC |
| **CD14** | cMo | Basophils |
| **CD16** | ncMo | pDC |
| **CD33** | cMo | B cells |
| **CD34** | HPC | Basophils |
| **CD35** | FcεRI^-^ cMo | NK cells |
| **CD36** | iMo | Basophils |
| **CD45*** | T cells | Basophils |
| **CD62L** | Basophils | Slan^+^ ncMo |
| **CD64** | FcεRI^-^ cMo | B cells |
| **CD117** | HPC | T cells |
| **CD141** | CD141^+^ myDC | Basophils |
| **CD163** | CD1c^+^ CD14^-^ CD5^-^ myDC | T cells |
| **CD192** | Basophils | Slan^+^ ncMo |
| **CD303** | pDCS | Basophils |
| **CD300e** | ncMo | Basophils |
| **FcERI** | Basophils | T cells |
| **HLA-DR** | iMo | Basophils |
| **Slan** | Slan^+^ ncMo | T cells |

* No negative population is available in the reference samples for this marker, therefore the NRP corresponded to a population with low expression levels of the marker evaluated.

***Abbreviations:*** *cMo, classical monocyte; HPC, hematopoietic precursor cells; iMo, intermediate monocyte; myDC, myeloid dendritic cell; ncMo, non-classical monocytes; NRP, negative reference population; pDC, plasmacytoid dendritic cells; PRP, positive reference population;*

.

**Supplementary Table 3: Comparative evaluation of the relative distribution of the different peripheral blood mononuclear cells (PBMC) populations identified in paired samples analyzed by spectral flow cytometry (SFC) and** **mass cytometry (MC).**

| **Populations** | **% of PBMC*** | | | **Correlation between platforms **** [Pearson’s rho values] | |
| --- | --- | --- | --- | --- | --- |
|  | **SFC** | **MC** | **Downsampled SFC (dSFC)** | **SFC *vs.* MC** | **dSFC *vs.* MC** |
| **Neutrophils** | 0.0001 (0.0 – 0.003) | 0.005 (0.0 – 0.1) | 0.0 (0.0 – 0.002) | 0.183 | -0.609 |
| **Basophils** | 0.9 (0.4 – 2.2) | 0.8 (0.4 – 2.3) | 0.9 (0.4 – 2.2) | 0.973 | 0.972 |
| **Monocytes (Mo)** | 21.0 (13.7 – 24.9) | 23.1 (11.7 – 28.1) | 21.0 (13.8 – 25.3) | 0.889 | 0.859 |
| **cMo** | 17.9 (12.7 – 22.6) | 21.6 (10.8 – 22.8) | 18.3 (12.8 – 23.1) | 0.861 | 0.862 |
| **CD62L^+^ FcεRI^-^** | 12.2 (7.4 – 15.5) | 10.2 (4.1 – 12.3) | 11.4 (6.1 – 13.1) | 0.854 | 0.973 |
| **CD62L^+^ FcεRI^+^** | 2.8 (0.3 – 7.5) | 3.8 (0.3 – 10.5) | 3.5 (1.0 – 10.5) | 0.944 | 0.993 |
| **CD62L^-^ FcεRI^-^** | 1.4 (1.2 – 5.7) | 2.4 (0.8 – 7.6) | 1.8 (1.0 – 5.9) | 0.743 | 0.857 |
| **CD62L^-^ FcεRI^+^** | 0.6 (0.2 – 1.1) | 0.5 (0.1 – 4.9) | 0.2 (0.1 – 0.6) | 0.555 | 0.978 |
| **iMo** | 0.6 (0.2 – 1.5) | 0.4 (0.1 – 1.0) | 0.3 (0.2 – 0.7) | 0.991 | 0.836 |
| **ncMo** | 0.5 (0.1 – 3.3) | 0.7 (0.1 – 4.4) | 0.7 (0.1 – 3.4) | 0.978 | 0.974 |
| **CD36^+^ Slan^-^** | 0.2 (0.1 – 1.2) | 0.3 (0.1 – 3.2) | 0.4 (0.1 – 1.7) | 0.981 | 0.976 |
| **CD36^-^ Slan^-^** | 0.1 (0.01 – 0.3) | 0.1 (0.02 – 0.4) | 0.1 (0.01 – 0.3) | 0.880 | 0.910 |
| **CD36^-^ Slan^+^** | 0.2 (0.005 – 0.9) | 0.1 (0.02 – 0.5) | 0.2 (0.01 – 0.8) | 0.967 | 0.965 |
| **CD36^+^ Slan^+^** | 0.05 (0.003 – 0.9) | 0.1 (0.02 – 0.3) | 0.06 (0.01 – 0.6) | 0.828 | 0.917 |
| **M-MDSC** | 0.2 (0.1 – 0.3) | 0.2 (0.08 – 0.2) | 0.1 (0.02 – 0.2) | -0.890 | 0.248 |
| **Dendritic cells (DC)** | 1.0 (0.3 – 1.7) | 1.2 (0.3 – 1.8) | 1.0 (0.4 – 1.7) | 0.865 | 0.917 |
| **CD1c^+^ myDC** | 0.8 (0.3 – 1.5) | 0.6 (0.3 – 1.2) | 0.7 (0.4 – 1.3) | 0.891 | 0.969 |
| **CD1c^+^ CD14^low^** | 0.1 (0.1 – 0.7) | 0.2 (0.1 – 0.3) | 0.2 (0.1 – 0.5) | 0.963 | 0.835 |
| **CD1c^+^ CD14^-^ CD5^-^** | 0.5 (0.1 – 0.8) | 0.3 (0.1 – 0.9) | 0.5 (0.1 – 0.6) | 0.965 | 0.897 |
| **CD1c^+^ CD14^-^ CD5^+^** | 0.1 (0.05 – 0.3) | 0.1 (0.04 – 0.2) | 0.1 (0.05 – 0.3) | 0.757 | 0.899 |
| **CD141^+^ myDC** | 0.05 (0.01 – 0.1) | 0.1 (0.01 – 0.1) | 0.05 (0.01 – 0.1) | 0.980 | 0.997 |
| **pDC** | 0.1 (0.004 -0.4) | 0.1 (0.03 – 0.6) | 0.1 (0.007 – 0.4) | 0.977 | 0.975 |
| **Axl^+^ DC** | 0.02 (0.002 – 0.03) | 0.02 (0.005 – 0.02) | 0.02 (0.005 – (0.02) | 0.880 | 0.932 |
| **CD34^+^ HPC** | 0.04 (0.02 – 0.08) | 0.03 (0.02 – 0.08) | 0.03 (0.01 – 0.07) | 0.946 | 0.923 |
| **DC precursors** | 0.003 (0.001 –0.006) | 0.02 (0.004 – 0.08) | 0.007 (0.005 – 0.03) | 0.459 | 0.810 |
| **Total lymphocytes** | 77.2 (72.5 – 84.6) | 74.8 (68.0 – 87.2) | 77.0 (72.3 – 84.8) | 0.875 | 0.875 |
| **T cells** | 58.9 (56.5 – 67.1) | 56.4 (53.2 – 63.1) | 58.9 (56.3 – 67.1) | 0.899 | 0.916 |
| **B cells** | 6.4 (3.9 – 13.4) | 8.1 (4.7 – 10.6) | 6.1 (4.1 – 13.4) | 0.880 | 0.866 |
| **NK cells** | 9.0 (4.1 – 19.3) | 9.8 (6.0 – 19.8) | 9.0 (4.1 – 19.3) | 0.665 | 0.666 |
| **Unclassified leukocytes** | 0.1 (0.0 – 0.7) | 0.2 (0.01 – 1.0) | 0.3 (0.2 – 0.9) | 0.939 | 0.841 |
| **% of populations with an rho ≥ 0.8** | | | | **76.6% (23/30)** | **90.0% (27/30)** |

Results expressed as **median percent values (range) or as **Pearson’s rho.

Populations showing a correlation factor (rho) of ≤0.800 are highlighted in grey.

Median (range) of total live PBMC evaluated: 2,663,810 (2,424,389 – 3,270,957) by SFC and 1,401,847 (1,207,092 – 1,695,400) by MC and downsampled SFC.

***Abbreviations:*** *Axl DC, Axl^+^ dendritic cell; DC, dendritic cell; DC precursor, CD100^+^ dendritic cell precursor; dSFC, samples measured on the spectral flow cytometer with data file downsampled to match the number of live leukocytes evaluated in the paired mass cytometry data file; cMo, classical monocyte; HPC, hematopoietic precursor cell; iMo, intermediate monocyte; MC, mass cytometry; myDC, myeloid dendritic cell; M-MDSC, monocytic myeloid-derived suppressor cell; ncMo, non-classical monocyte; pDC, plasmacytoid dendritic cell; SFC, spectral flow cytometry.*
