# Supplementary figures and images for: Performance of spectral flow cytometry and mass cytometry for the study of innate myeloid cell populations
Source: Front Immunol. 2023 May 19;14:1191992. doi: 10.3389/fimmu.2023.1191992 (PMC10235610; doi:10.3389/fimmu.2023.1191992)

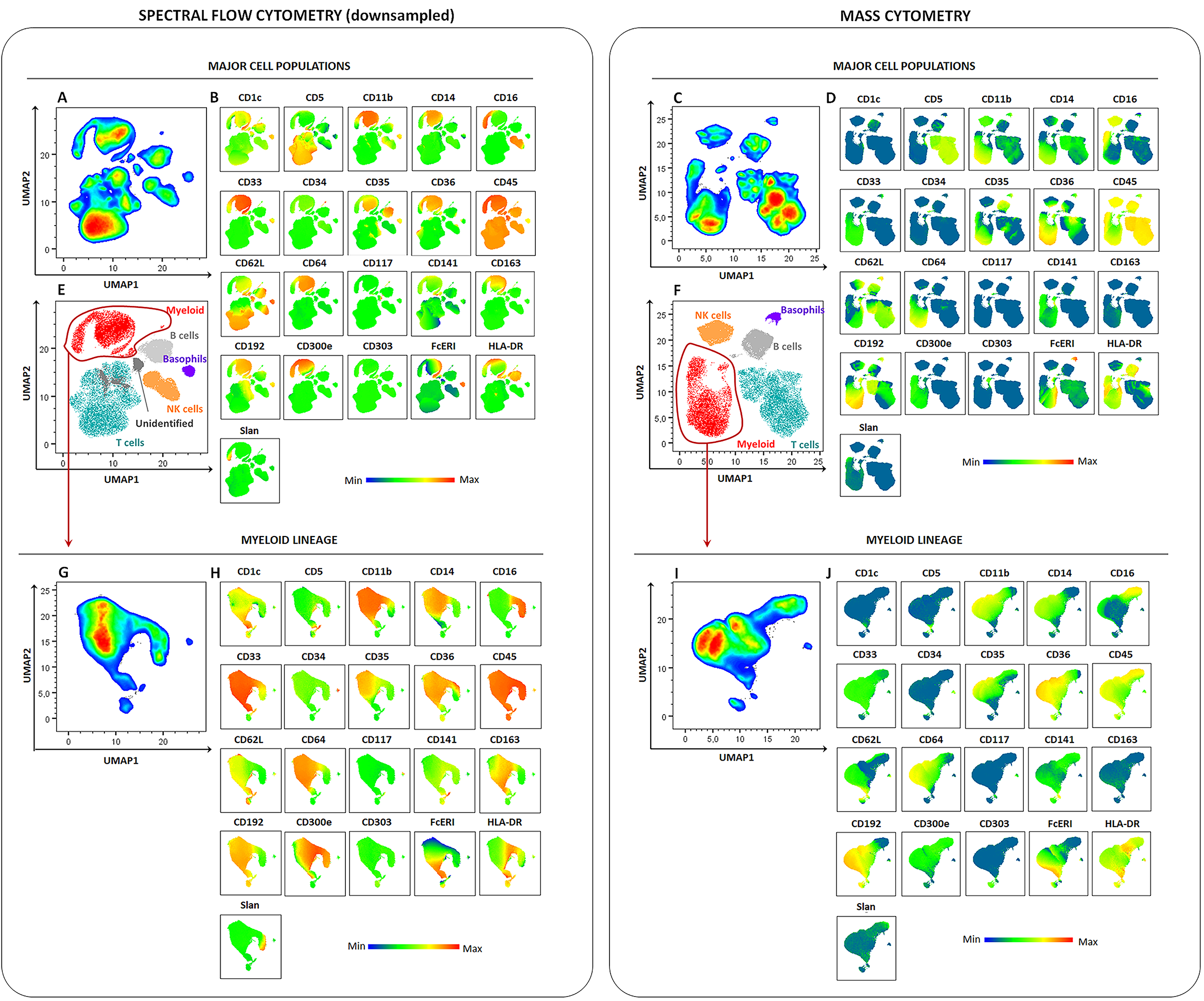

Supplement: Supplementary file 2 [file Image_1.tif]

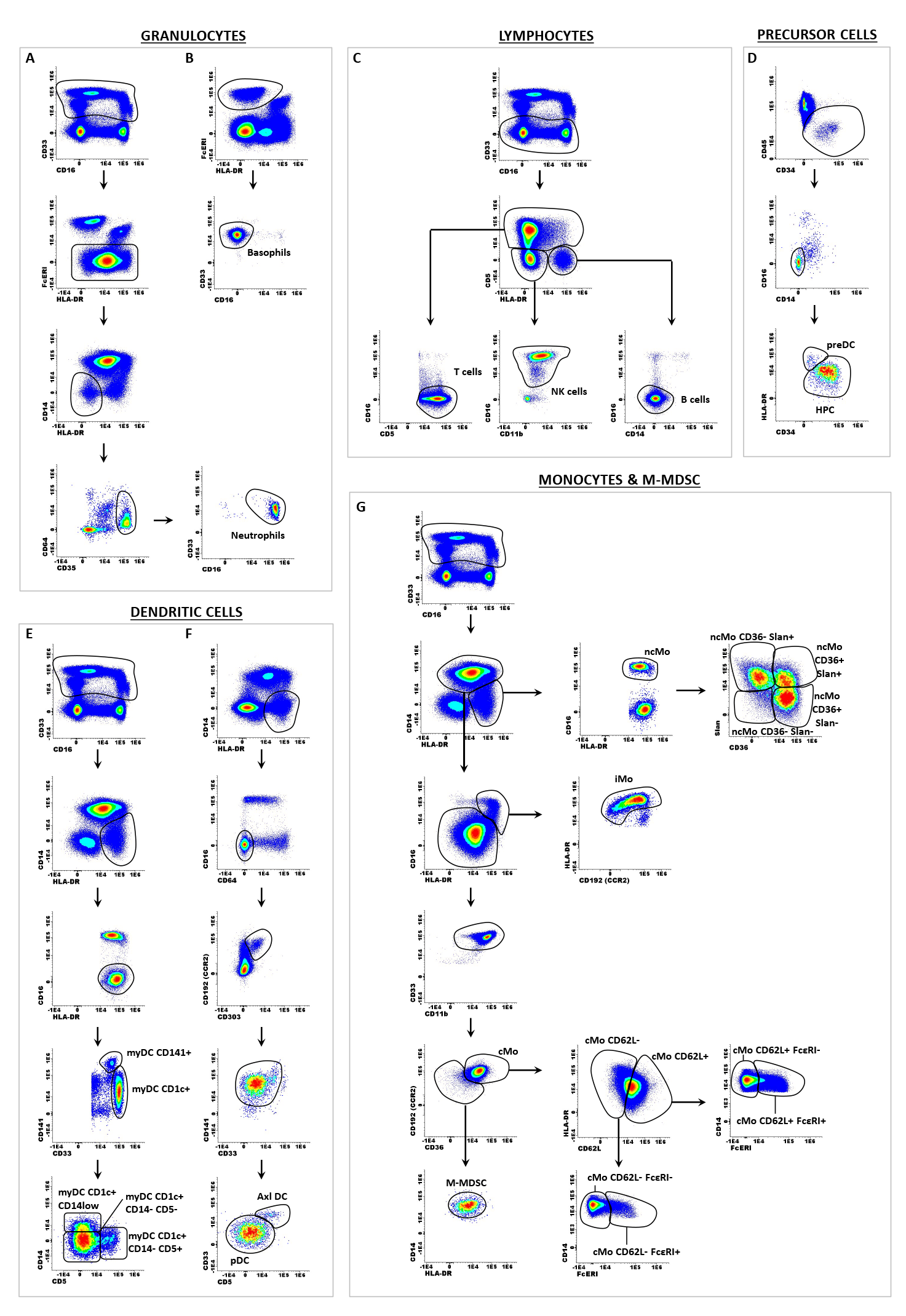

Supplement: Supplementary file 3 [file Image_2.tif]

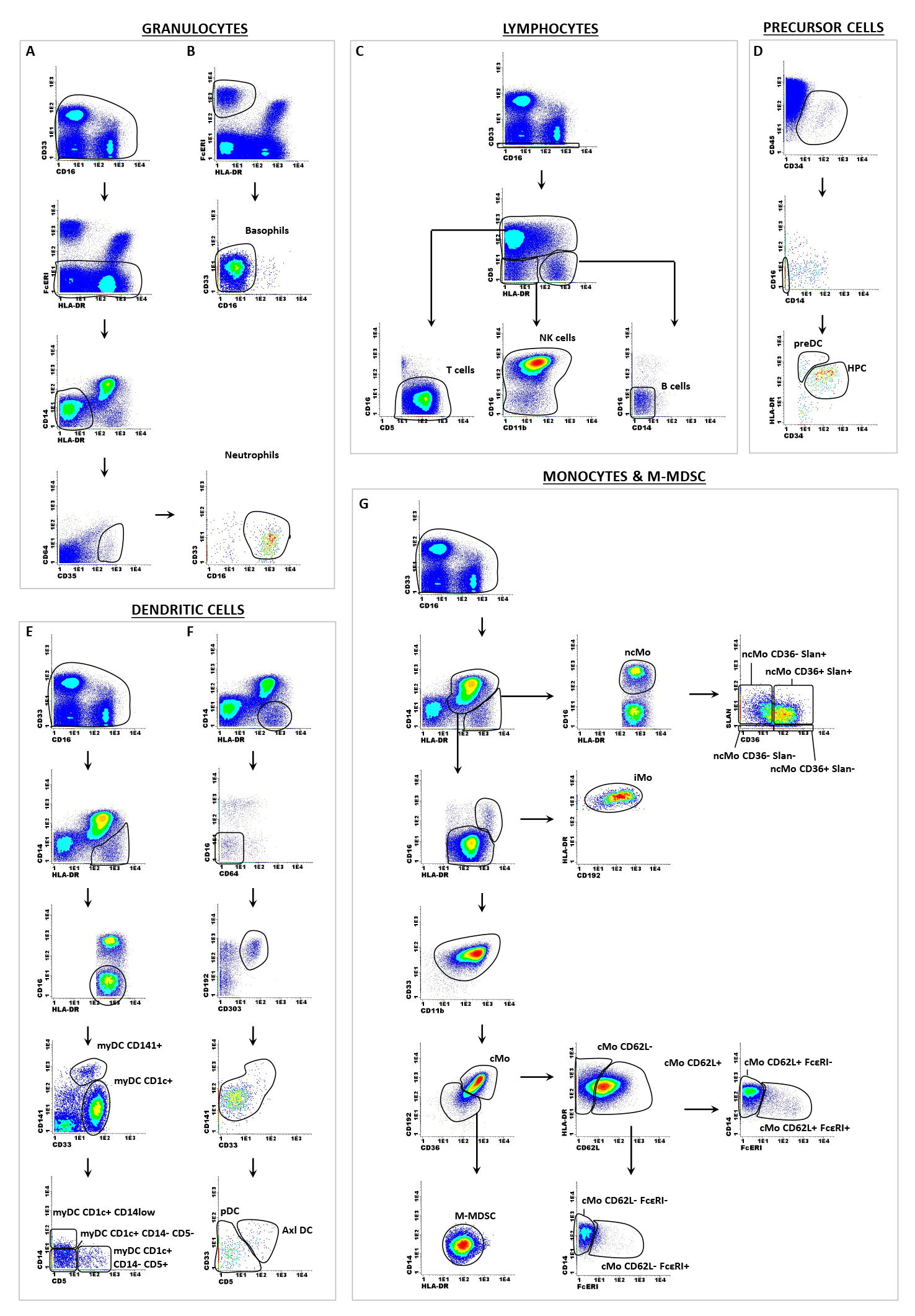

Supplement: Supplementary file 4 [file Image_3.tif]

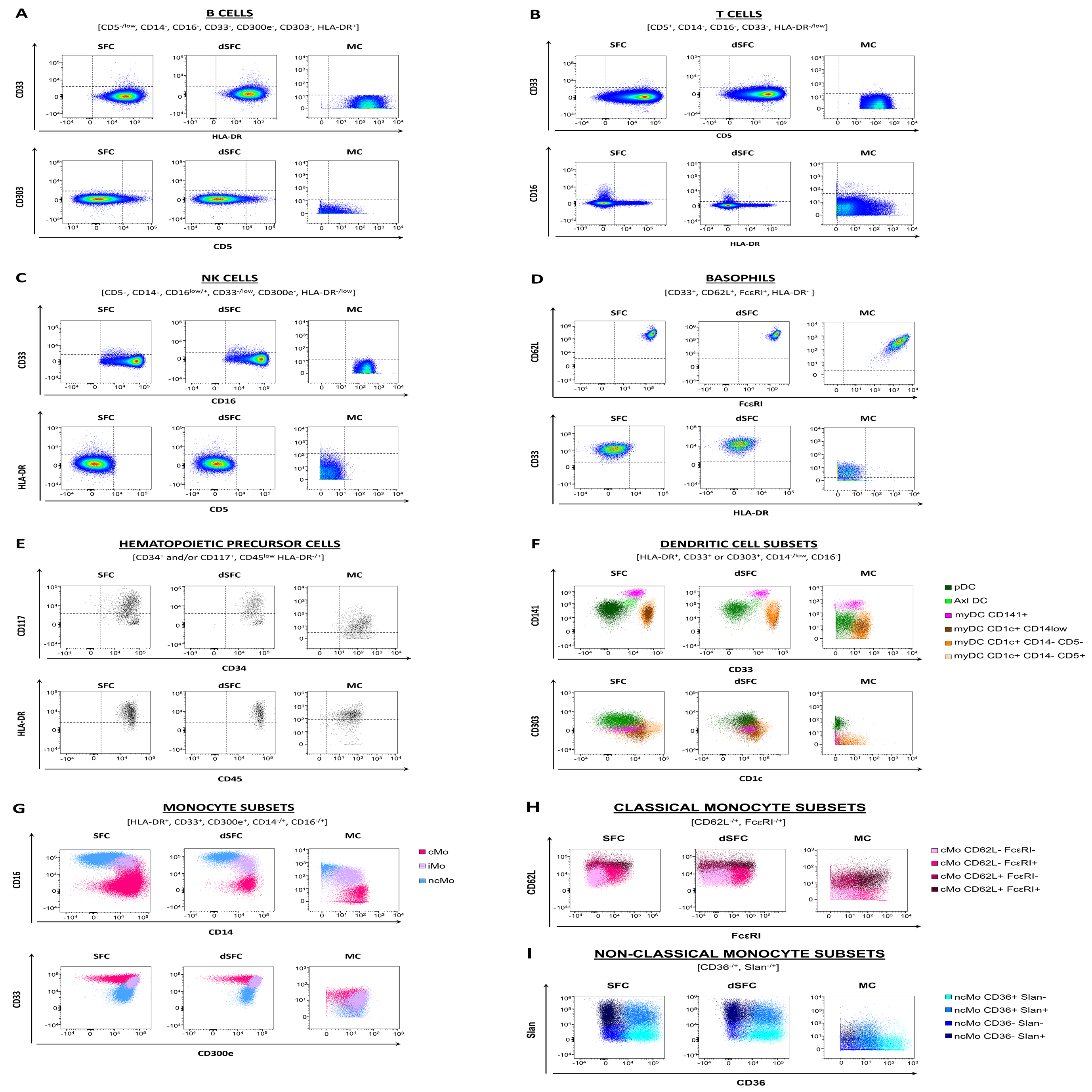

Supplement: Supplementary file 5 [file Image_4.tif]

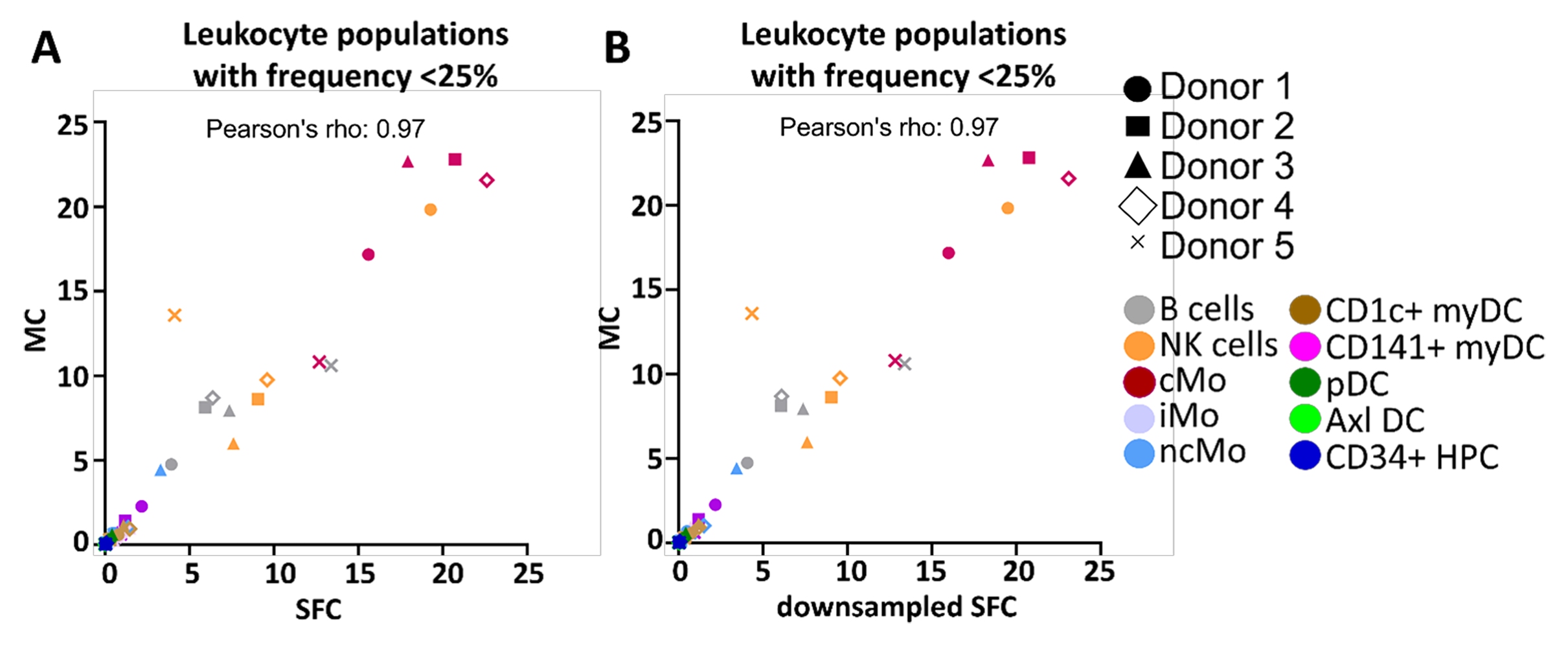

Supplement: Supplementary file 6 [file Image_5.tif]

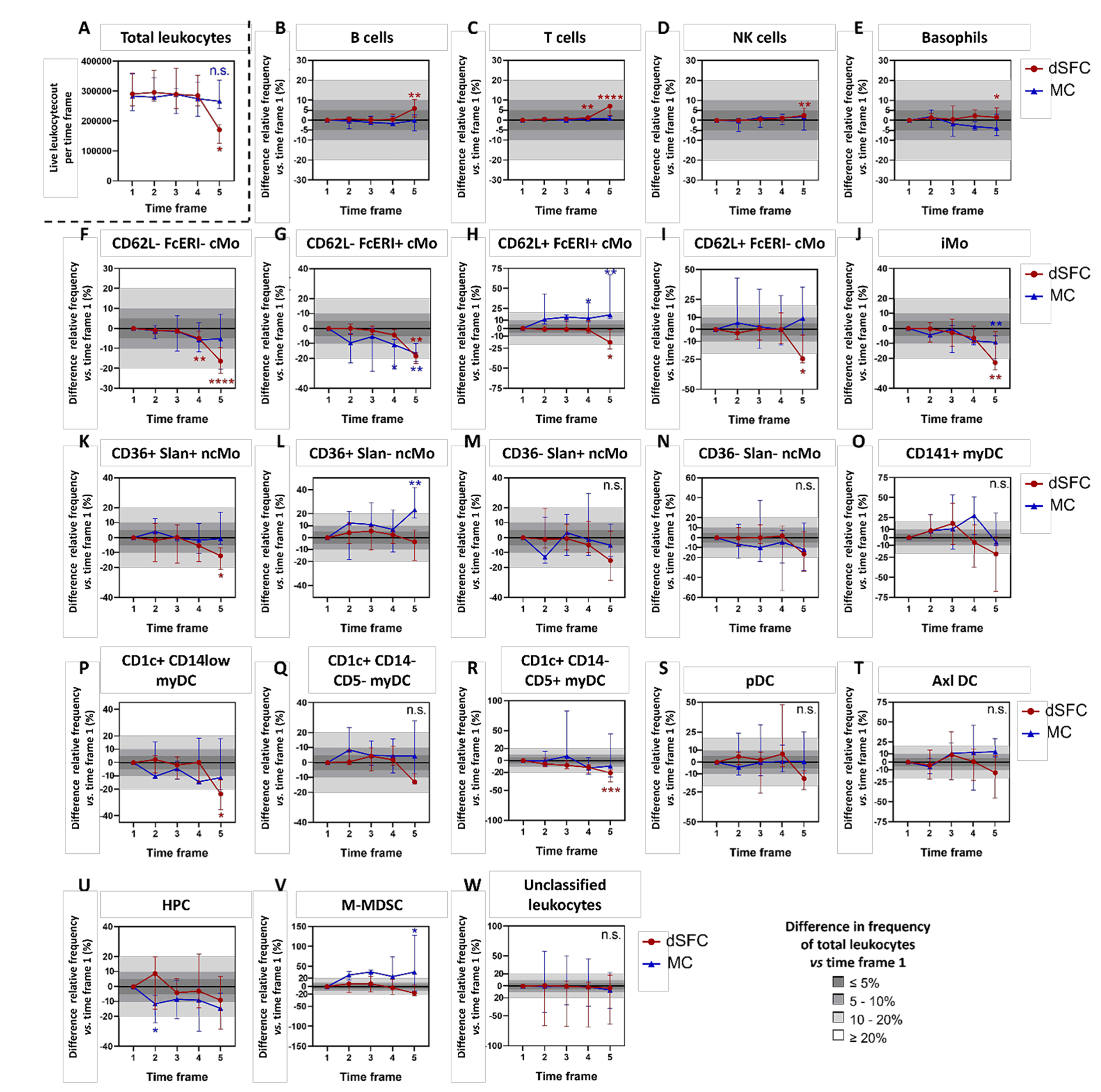

Supplement: Supplementary file 7 [file Image_6.tif]
